# Effects of electric field direction on TMS-based motor cortex mapping

**DOI:** 10.1162/IMAG.a.1211

**Published:** 2026-04-21

**Authors:** Ying Jing, Ole Numssen, Gesa Hartwigsen, Thomas R. Knösche, Konstantin Weise

**Affiliations:** Methods and Development Group Brain Networks, Max Planck Institute for Human Cognitive and Brain Sciences, Leipzig, Germany; Research Group Cognition and Plasticity, Max Planck Institute for Human Cognitive and Brain Sciences, Leipzig, Germany; Complex Networks and Brain Dynamics Group, Institute of Computer Science of the Czech Academy of Sciences, Prague, Czech Republic; TMS Group, Department of Neuroscience and Biomedical Engineering, Aalto University, Finland; Wilhelm Wundt Institute for Psychology, Leipzig University, Leipzig, Germany; Institute of Biomedical Engineering and Informatics, Technische Universität Ilmenau, Ilmenau, Germany; Institute of Electrical Energy Technology, Leipzig University of Applied Sciences, HTWK, Leipzig, Germany

**Keywords:** transcranial magnetic stimulation, TMS mapping, electrical field modeling, average response model, motor cortex

## Abstract

Transcranial magnetic stimulation (TMS) induces an electric field (E-field) that drives neuronal activation, but the optimal model for predicting cortical responses remains unclear. Traditional TMS motor mapping typically relies on the E-field magnitude or its normal component as a proxy for excitability, overlooking the influence of neuronal morphology and orientation. In this study, we aimed to refine TMS motor mapping by incorporating an average response model that accounts for both E-field magnitude and directional sensitivity. We conducted a regression-based TMS mapping experiment in 14 participants to identify cortical origins of motor-evoked potentials (MEPs) from the first dorsal interosseous (FDI) muscle. Firing thresholds were estimated for excitatory neurons in cortical layers 2/3 and 5, and regression was performed between MEPs and three E-field quantities: the E-field magnitude (magnitude model), the normal component of E-field (cosine model), and an effective E-field that adjusts magnitude by orientation-specific thresholds (neuron model). Models were compared based on regression fit, convergence speed, and functional validation using optimized coil placements tested in 10 additional participants. Results showed that the magnitude and neuron models performed similarly and robustly, whereas the cosine model explained significantly less variance, required more TMS pulses for stable mapping, and produced the weakest MEPs in validation. These findings suggest that while directional sensitivity plays a role, E-field magnitude remains the dominant factor in motor cortex activation.

## 1 Introduction

Since the first demonstration in 1985 (Barker et al., 1985), transcranial magnetic stimulation (TMS) has become a widely used technique for non-invasively modulating brain activity in vivo. By transiently altering brain function, TMS enables causal mapping of brain structure-function relationships, using measures such as motor-evoked potentials (MEPs) to identify cortical sites responsible for specific functions (Gunduz et al., 2020; Van De Ruit et al., 2015). This capability has been widely applied in pre-surgical target selection and functional assessment following neurological injuries (Seynaeve et al., 2019; Sondergaard et al., 2021).

While TMS is FDA-cleared for cortical mapping (Cohen et al., 2022) and the treatment of several psychiatric disorders (George et al., 2009; McNamara et al., 2001), it is still largely unknown which neuron structures are primarily excited by a TMS pulse and how this localized activation leads to behavioral or psychological outcomes (Romero et al., 2019). The wide spatial distribution of TMS-induced electric fields (E-fields) across large cortical areas further complicates the identification of the effective target (Bijsterbosch et al., 2012). Consequently, TMS studies often show small effect sizes (Beynel et al., 2019) and exhibit considerable variability both between and within subjects (Caulfield & Brown, 2022; Numssen et al., 2023), limiting the general efficacy of TMS in both basic research and clinical applications (Hartwigsen & Silvanto, 2023). This observed variability in TMS effects is attributed to a complex interplay of various factors, including physiological parameters across macro-, meso-, and microlevels (e.g., gyrification pattern, spatial organization of cortical neurons, neuron types, respectively), methodological parameters (e.g. pulse shape, coil geometry, stimulator intensity), and cognitive factors (e.g., response strategies, cognitive brain state) (Numssen et al., 2024; Williams et al., 2024).

Although numerical simulations of TMS-induced E-fields have improved cortical mapping, the precision of these models remains debated (Dannhauer et al., 2024). Early approaches assumed that the cortical site of maximum E-field strength corresponds to the functional hotspot (Aonuma et al., 2018; Opitz et al., 2013; Thielscher & Kammer, 2002), but the strongest E-field does not necessarily coincide with the neurons generating responses (Weise et al., 2020). Linear models relating E-field strength to MEP amplitude (Matthaeus et al., 2008) also proved insufficient, since cortical response to stimulation is inherently non-linear: MEPs appear only after surpassing a threshold and plateau as E-field strength continues to increase (Devanne et al., 1997; Goetz et al., 2014; Malcolm et al., 2006). To address these limitations, we recently proposed a non-linear, regression-based TMS mapping method (Numssen et al., 2021; Weise et al., 2020) that revealed a strong, sigmoid-like relationship between local E-field magnitude and MEPs at the cortical muscle representation, which is primarily located on the crowns and rims of the precentral gyrus (Siebner et al., 2022; Vetter et al., 2025; Weise, Numssen, et al., 2023).

Critically, the response of neurons to TMS is not solely influenced by the magnitude of induced E-field, but also by complex interactions between E-fields and neuron morphology, as well as their spatial organization within cortical layers (Di Lazzaro & Rothwell, 2014; Hannah & Rothwell, 2017; Reijonen et al., 2020). The direction of the induced current is particularly important, as axons are activated by differences in potential along their length. Early experimental studies revealed that single neurons are more responsive to current oriented longitudinally rather than transversely across it (Ranck Jr, 1975; Rushton, 1927). This led to the well-known cortical column cosine model (Fox et al., 2004), which assumes that the depolarization threshold of axons is inversely proportional to the cosine of the angle between the external E-field and the principal axis of the neuron. However, this model overemphasizes the normal field component and oversimplifies the complex orientations of neurons. Recent studies utilizing realistic simulations of neuronal geometry have revealed that axons often exhibit complex branching, contributing to a nuanced directional sensitivity (Aberra et al., 2020, 2023; Weise, Worbs, et al., 2023).

To address this, a computationally efficient model was developed that determines the averaged firing thresholds of neurons from different cortical layers by subjecting various types of cortical neurons to TMS-induced E-fields with different angles, intensities, pulse waveforms, and field gradients along the somato-dendritic axis (Weise, Worbs, et al., 2023). The major advantage of this model is that it allows for a straightforward transformation from macroscopic E-field topographies to neuron firing thresholds without sacrificing modeling accuracy. The firing threshold of any given cortical location at any layer can be determined using precomputed look-up tables and interpolators, which account for the estimated polar angle θ between the E-fields and axons, as well as the field gradient $\Delta |\;\;\tilde{E}\;|$ at a particular cortical location. This firing threshold quantifies the strength of the E-field required to elicit an action potential. By scaling the raw E-field magnitude |E| with the firing threshold, the effective E-field $E_{eff}$ , which accounts for neuronal response characteristics, can be estimated to provide a more precise estimation of TMS effects.

In this study, we incorporated this novel neuronal response model into our regression-based TMS mapping method to refine the estimation of cortical muscle representations. This mapping method, referred to as the neuron-enhanced model (or simply the *neuron model*), uses the effective E-field $E_{eff}$ to estimate neuronal response to stimulation. The neuron model was evaluated in comparison to two previous models: (1) the classical *magnitude model*, as used by Numssen et al. (2021), which considers the magnitude of the E-field |E| as a proxy for neuronal activation, and (2) the cortical column cosine model, or simply the *cosine model*, assuming that axonal depolarization is driven primarily by the normal component of the E-field relative to the cortical surface ($\left|E_{⊥}\right|$). Unlike these two simplified models, the *neuron model* ($E_{eff}$ ) scales the E-field’s magnitude with the local firing threshold in L2/3 and L5, as neurons in these layers primarily project to the spinal cord and, thus, are the origin of MEPs (Aberra et al., 2020; Wilson et al., 2021). These three models constitute a set of *neuronal response models*, each simulating how neurons respond to E-fields (see Fig. 2D for an illustration of these three E-field quantities).

In summary, while the *magnitude model* (|E|) has proven effective in TMS motor mapping, it ignores neuronal orientation and may therefore miss subtle, direction-dependent contributions to cortical activation. In contrast, the *cosine model* ($\left|E_{⊥}\right|$) overemphasizes the normal component of the E-field in determining neuronal response to stimulation and can lead to biased estimations. To improve mapping accuracy, we introduced the *neuron model* ($E_{eff}$ ), which integrates both E-field magnitude and directional sensitivity, thus providing a more biophysically realistic description of how TMS interacts with cortical neurons. By directly comparing the neuron model with the simpler magnitude and cosine models, we aimed to determine whether the neuron model can increase the accuracy of TMS-based cortical mapping, or whether the magnitude model already captures the dominant determinants of neuronal activation, implying a practical precision limit for current mapping methods.

**Fig. 1.**
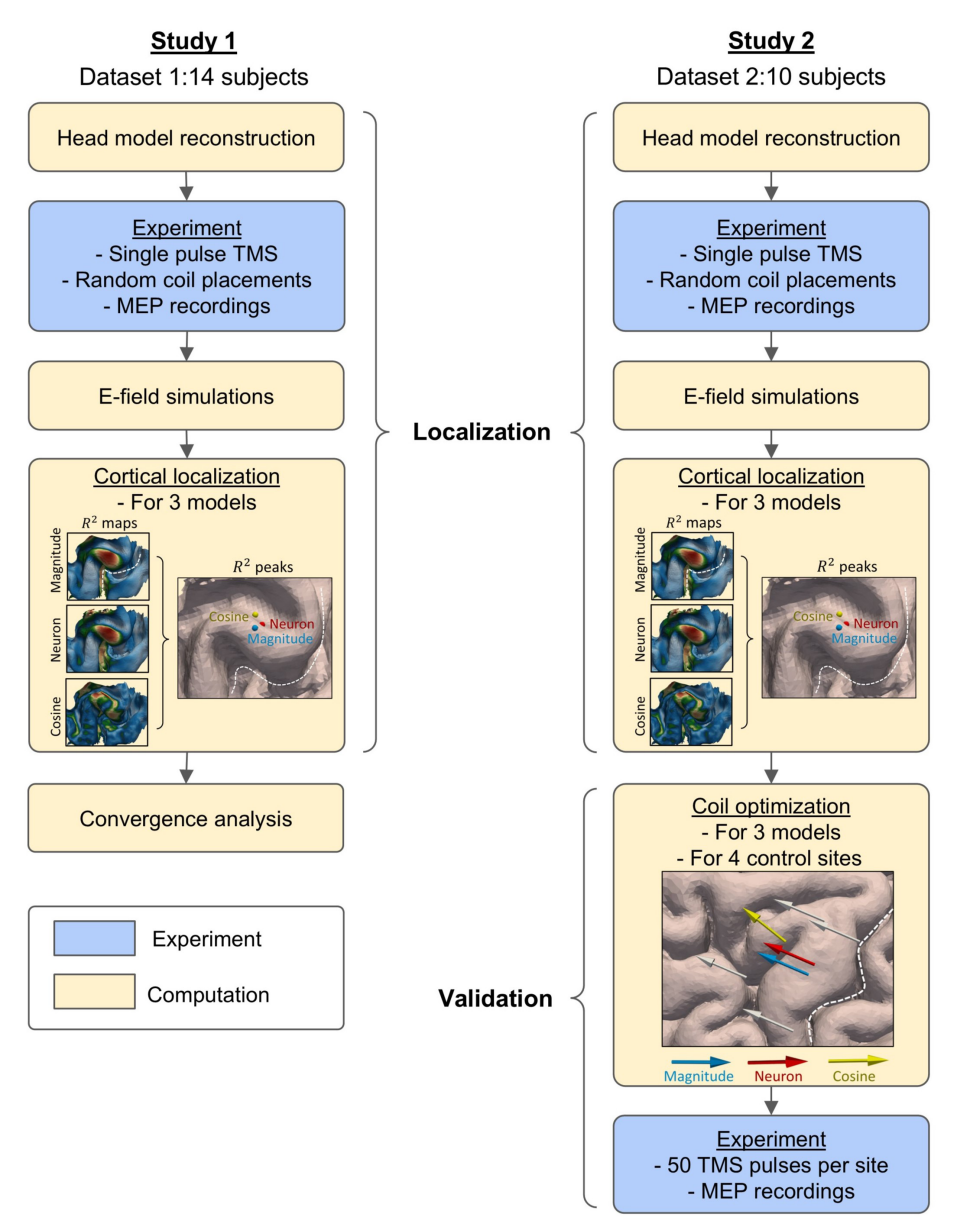
Overview of the study design. Study 1 (left) was based on Dataset 1 with 14 participants (Numssen et al., 2021). The TMS localization experiment involved four main steps: 1) Head model reconstruction. Individual head models were reconstructed from MRI data and used for numerical simulations of induced electric fields (E-fields). 2) Experiment. For each participant, TMS was applied over the left primary motor cortex (M1) with 900–1000 pulses from random coil positions and orientations, and motor evoked potential (MEP) amplitudes were recorded. 3) E-field simulations. Simulated E-fields were computed for each TMS pulse using the individual head models. 4) Cortical localization. At each cortical element within the region of interest, three different E-field quantities (total E-field magnitude |E|, effective E-field $E_{eff}$ , and the normal component of the E-field $\left|E_{⊥}\right|$) were regressed against the MEP amplitudes. The resulting goodness-of-fit ($R^{2}$) maps indicate the most probable cortical origin of MEPs. A subsequent Convergence analysis was then performed to compare localization consistency across models. Study 2 (right) included Dataset 2 with 10 newly recruited participants, serving as an independent validation. The same TMS localization procedure as in Study 1 was applied to identify individual cortical hotspots, followed by a validation phase comprising: 1) Coil optimization. The optimal coil placements predicted by magnitude, neuron, and cosine models were computed for each participant. 2) Validation Experiment. At each coil placement, 50 pulses were delivered, and MEP amplitudes were compared across three models and four control sites to evaluate which model best identified the cortical origin of the MEPs.

We hypothesized that the neuron model would provide a more nuanced representation of neuronal activation. However, since the neuron model predicts only moderate directional sensitivity across all neuron types and the magnitude model already captures the dominant influence of E-field magnitude, we expected the magnitude model to align more closely with the neuron model than the cosine model, which assumes an overly strong directional sensitivity. To test these hypotheses, we conducted a validation session following an initial regression-based localization session (Fig. 1). In this validation session, TMS was performed using optimal coil placements based on all three models to determine which model yields the highest MEPs, that is, which model optimally locates the muscle representation. Additionally, we performed an extensive convergence analysis to test which model requires the fewest TMS pulses to obtain stable mapping results.

## 2 Methods

### 2.1 Subjects

Dataset 1 was reused from Numssen et al. (2021) and consists of 14 healthy, right-handed participants (seven females, aged 21–38 years). For a detailed description of subject recruitment, please refer to the original paper. All participants completed a TMS localization experiment in 2020, and this dataset was analyzed for both TMS localization and convergence analysis (Study 1, Fig. 1).

To account for potential changes in motor function over the 4-year interval, we decided to recruit 10 new participants, who completed both a TMS localization session (see Section 2.4) and a validation session (see Section 2.5) approximately 1 week apart. Dataset 2 (Study 2) includes these 10 healthy, right-handed participants (five females, aged 24–43 years), with a mean laterality index of 71 (SD = 15) as measured by the Edinburgh Handedness Inventory. None of these participants had contraindications to TMS, were on regular medication, or had a history of psychiatric or neurological diseases.

All participants of both datasets provided written informed consent to participate prior to the experiment. The studies were approved by the local Ethics committee of the Medical Faculty of Leipzig University. The research was conducted in accordance with the principles of the Declaration of Helsinki and local statutory requirements.

### 2.2 Imaging

Magnetic resonance imaging (MRI) data, including structural and diffusion-weighted scans, were acquired using a 3 Tesla Siemens Skyra fit scanner equipped with a 32-channel head coil. These scans were used for segmenting head tissues in E-field calculations and for neuronavigation during TMS. For structural imaging, T1- and T2-weighted images were obtained with the following parameters: T1 MPRAGE sequence with 176 sagittal slices, matrix size = 256 × 240, voxel size = 1 × 1 × 1 mm^3^, flip angle = 9°, and TR/TE/TI = 2300/2.98/900 ms; T2-weighted sequence with 192 sagittal slices, matrix size = 512 × 512, voxel size = 0.488 × 0.488 × 1 mm^3^, flip angle = 120°, and TR/TE = 5000/395 ms. A diffusion-weighted MRI (dMRI) scan was performed to estimate conductivity anisotropy in gray and white matter. This scan included 88 axial slices with a matrix size of 128 × 128, voxel size = 1.719 × 1.719 × 1.7 mm^3^, TR/TE = 80/7000 ms, flip angle = 90°, 67 diffusion directions, and a b-value of 1000 s/mm^3^. If a participant had an existing MRI scan in the image database that was less than 2 years old, it was used instead of acquiring a new scan.

### 2.3 Electromyography

Electromyograms (EMGs) from the right first dorsal interosseous (FDI) muscle were recorded using pairs of Ag-AgCl surface electrodes in a standard belly-tendon montage (Kleim et al., 2007). The raw signal was amplified with a patient amplifier system (D-360, DigitimerLtd., UK, Welwyn Garden City), bandpass filtered from 10 Hz to 2 kHz, and recorded with an acquisition interface (Power1401 MK-II, CED Ltd., UK, Cambridge) at a 4 kHz sampling rate using Signal software (CED Ltd., version 4.11). MEPs were calculated as peak-to-peak amplitudes within a time window of 18 to 35 ms after the TMS pulse.

### 2.4 TMS localization

#### 2.4.1 TMS protocol

TMS pulses were applied using a MagPro X100 stimulator (MagVenture, firmware Version 7.1.1) with an MCF-B65 figure-of-eight coil. A TMS navigation system (Localite software, Germany, Sankt Augustin; camera: Polaris Spectra, NDI, Canada, Waterloo) was employed to guide motor threshold (MT) hunting and to record the coil’s orientations and positions in real-time during the localization experiment.

We manually measured the resting MT (rMT) for the FDI muscle, with earplugs provided to prevent hearing damage during TMS. The stimulation site was first determined by locating the hand knob on the precentral gyrus (Yousry et al., 1997) based on individual T1 images. The motor hotspot was then identified by systematically varying the coil’s tilt, rotation, and location until a placement was found that elicited stable MEPs. An estimate of the rMT was determined at the motor hotspot as the lowest stimulator intensity required to produce MEPs with an amplitude of at least 50 µV in at least 5 out of 10 consecutive TMS pulses (Rothwell et al., 1999).

In Dataset 1, we utilized the data described by Numssen et al. (2021). In brief, to localize the muscle representation of the FDI, 900–1100 single biphasic TMS pulses were applied at 150% rMT with an inter-stimulus interval (ISI) of 5 s to the left motor area. For each TMS pulse, coil position and orientation were randomly selected within a 3 cm radius and the angles to approximately ± 60° in relation to the optimal coil placement (Fig. 3). The purpose of this randomization was to increase E-field variability and minimize cross-correlations between induced E-fields. All coil placements and corresponding MEPs from the FDI muscle were recorded.

Given that participants’ motor functions might have slightly changed after 4 years, we recruited 10 new subjects for Dataset 2 to perform the localization experiment again. We followed the same stimulation protocol as described above but with only 500 random TMS pulses. As shown in our convergence analysis (see Section 3.2), a minimum of 300 pulses was sufficient to achieve stable localization results.

#### 2.4.2 E-field simulation

E-fields induced for each TMS pulse were computed using SimNIBS v4.1.0 (Saturnino et al., 2019; Thielscher et al., 2015) with high-resolution anisotropic finite element models (FEMs).

To simulate E-fields, individual head models were first reconstructed via the CHARM pipeline (Head model reconstruction), which integrates FreeSurfer outputs for a more accurate representation of smaller sulci in the head meshes (Puonti et al., 2020). A refined region of interest covering the somatosensory cortex (BA1, BA3), primary motor cortex M1 (BA4), and dorsal premotor cortex PMd (BA6) was defined based on the fsaverage template to improve the numerical resolution around the ROI (Numssen et al., 2021). The final head models were composed of approximately 0.95 × 10^6^ nodes and 5.49 × 10^6^ tetrahedra (average volume: approximately 0.94 mm^3^ in the cortex, and 0.05 mm^3^ in the refined cortical ROI in the motor area). Six tissue types were included with standard conductivity estimates: white matter ($\sigma_{WM}$ = 0.126 S/m), gray matter ($\sigma_{GM}$ = 0.275 S/m), cerebrospinal fluid ($\sigma_{CSF}$ = 1.654 S/m), bone ($\sigma_{B}$ = 0.01 S/m), skin ($\sigma_{S}$ = 0. 465 S/m), and eyeballs ($\sigma_{EB}$ = 0.5 S/m) (Thielscher et al., 2015). WM and GM were assigned anisotropic conductivities, while the four other tissues were treated as isotropic.

Given that the magnitude and cosine models consider GM as a uniform entity and disregard the cytoarchitectonic differences across cortical layers, we first calculated E-fields only at the midlayer between GM and WM surfaces to avoid boundary effects of the E-field due to conductivity discontinuities. The E-field interpolation employs the super-convergent patch recovery method to determine the E-field at the GM center via linear interpolation (Saturnino et al., 2019).

To incorporate detailed geometric information across different cortical layers, we added cortical layers to the motor cortex ROI in each head model. These layers were defined by their normalized depths, ranging from 0 (gray matter surface) to 1 (white matter surface), based on estimates from primate motor cortex slices (García‐Cabezas & Barbas, 2014). The normalized depths for the centers of these layers are 0.06 for layer 1, 0.4 for layers 2/3, 0.55 for layer 4, 0.65 for layer 5, and 0.85 for layer 6 (Aberra et al., 2020; Weise, Worbs, et al., 2023). We linearly interpolated the positions of these layers between the white and gray matter surfaces using the vertex positions of the two surfaces. E-fields in layer 2/3 and layer 5 were then interpolated using SimNIBS, as they were for the midlayer. The number of ROI elements was about 0.95 × 10^4^ at the midlayer, and 2.78 × 10^4^ for layer 2/3 and layer 5.

#### 2.4.3 Firing threshold and effective E-field calculation

To account for neuronal excitability in TMS mapping, we estimated the firing threshold of neurons, which is defined as the minimum E-field intensity that elicits action potentials, using the average threshold model (Weise, Worbs, et al., 2023). This model provides pre-computed look-up tables for neuron firing threshold across a wide range of neuronal types and spatial orientations.

The neuron morphologies used in this average threshold model were extended from the compartmental models developed by Aberra et al. (2020), based on data from the Blue Brain Project (Markram et al., 2015). As the original morphologies were from rodents, further modifications were made to approximate human-like neuron features following procedures described in Aberra et al. (2018). The simulated neurons were then exposed to E-fields from different directions and strengths to determine the thresholds for each cell across all possible E-field configurations. The average threshold model was derived by averaging these thresholds over all compartment models and azimuthal orientations. The model’s accuracy was validated by comparing its results with computationally intensive reference simulations, which utilized a high-resolution, realistic head model containing a large number of neurons located within the motor cortex (Weise, Worbs, et al., 2023).

**Fig. 2.**
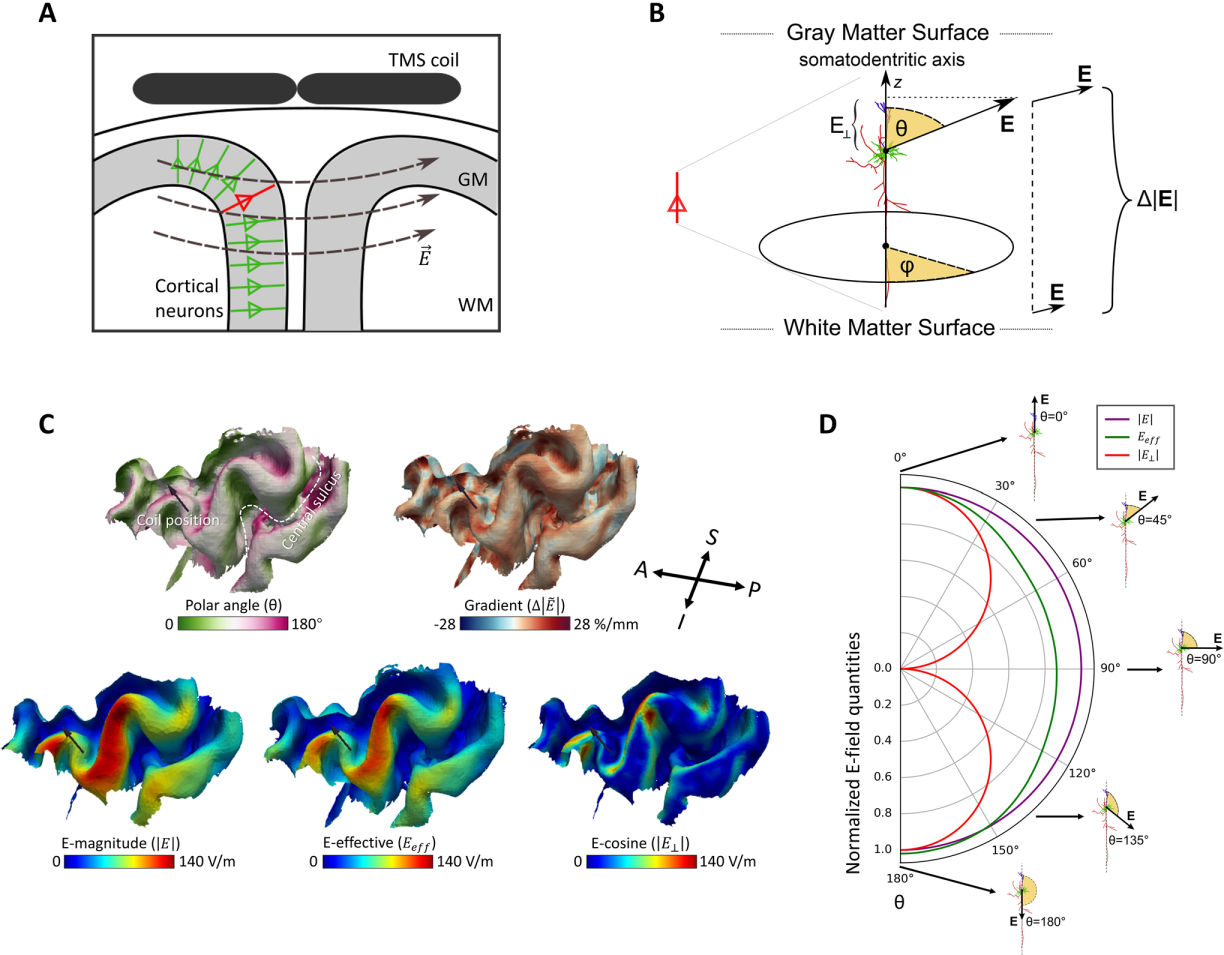
The three neuronal response models are based on different E-field quantities. (A) TMS-induced E-fields (E-fields) are oriented perpendicular to cortical neurons on the gyral crown but can run parallel to sulcal neurons. TMS: transcranial magnetic stimulation; GM: gray matter; WM: white matter; $\vec{E}$ : E-field vector, representing both the magnitude and direction of the induced e-field. (B) An example neuron exposed to the E-field. θ: the polar angle between the E-field and the somato-dendritic axis; φ: the E-field direction in the horizontal plane perpendicular to the somato-dendritic axis; Δ|  E | : The gradient of the E-field magnitude between gray and white matter surfaces; $E_{⊥}$: The normal component of the E-field along the somato-dendritic axis. The cosine model assumes that neurons respond exclusively to the magnitude of this component $\left|E_{⊥}\right|$. (C) Allocation of polar angle θ and gradient $\Delta |\;\;\tilde{E}\;|$ on layer 5 (L5), E-field magnitude |E| on gray matter midlayer, effective E-field $E_{eff}$ on L5, and $\left|E_{⊥}\right|$ on gray matter midlayer in one example TMS pulse. The tail of the arrow represents the coil position, and its direction represents coil orientation. A: anterior; P: posterior; S: superior; I: inferior. (D) The different neuronal response models (magnitude model |E|, neuron model $E_{eff}$ , and consine model $\left|E_{⊥}\right|$) yield different neuronal stimulation (radial axis) across θ from 0° to 180° at $\Delta |\;\;\tilde{E}\;|$ = 0.

To quantify the firing threshold of any given elements within the cortical ROI, we first calculated two key parameters for layer 2/3 and layer 5: i) polar angle θ: the relative angle between induced E-fields and the surface normal (somato-dendritic axis) of the cortical layers, ranging from 0° to 180°; ii) gradient $\Delta |\tilde{E}|$ : the relative change of the E-field magnitude along the somato-dendritic axis per unit length in %/mm (Fig. 2). To compute this, we first extracted the E-field magnitude at a normalized depth of 10% of the distance between the current layer and the GM or WM surface. The gradient is then determined by comparing the E-field at two adjacent points along this axis and calculating the rate of change per unit length. Firing thresholds for each element in the ROI across layers were then determined using the average threshold model, which provides a look-up table that defines the firing threshold in relation to the polar angle θ and gradient $\Delta |\tilde{E}|$ .

The normalized firing threshold $S\left(\theta ,\Delta |\tilde{E}|\right)$ is then obtained by dividing the original firing threshold at a given polar angle θ and gradient $\Delta |\tilde{E}|$ by its reference value at θ=0 and $\Delta |\tilde{E}|=0$ :

$$S\left(\theta ,\Delta |\tilde{E}|\right)=\frac{Threshold\left(\theta ,\Delta |\tilde{E}|\right)}{Threshold\left(0,\;0\right)}$$

This normalized firing threshold defines a measure of neuronal sensitivity to the induced E-field. S>1 indicates that neurons in that region are less excitable, requiring a stronger E-field to reach the firing threshold. However, S<1 suggests that neurons are more excitable that a weaker E-field is sufficient to elicit activation.

Subsequently, we calculated the effective E-field ($E_{eff}$ ) for each element in the ROI by dividing the E-field magnitude (|E|) by the normalized firing threshold, which depends on the polar angle θ and the gradient $\Delta |\tilde{E}|$ :

$$E_{eff}=\frac{\left|E\right|}{S\left(\theta ,\Delta |\tilde{E}|\right)}$$

By scaling |E| with the normalized firing threshold, the $E_{eff}$ (used in the neuron model) quantifies the stimulation strength in a way that accounts for the intrinsic excitability of neurons across different cortical layers.

#### 2.4.4 Cortical localization

**Fig. 3.**
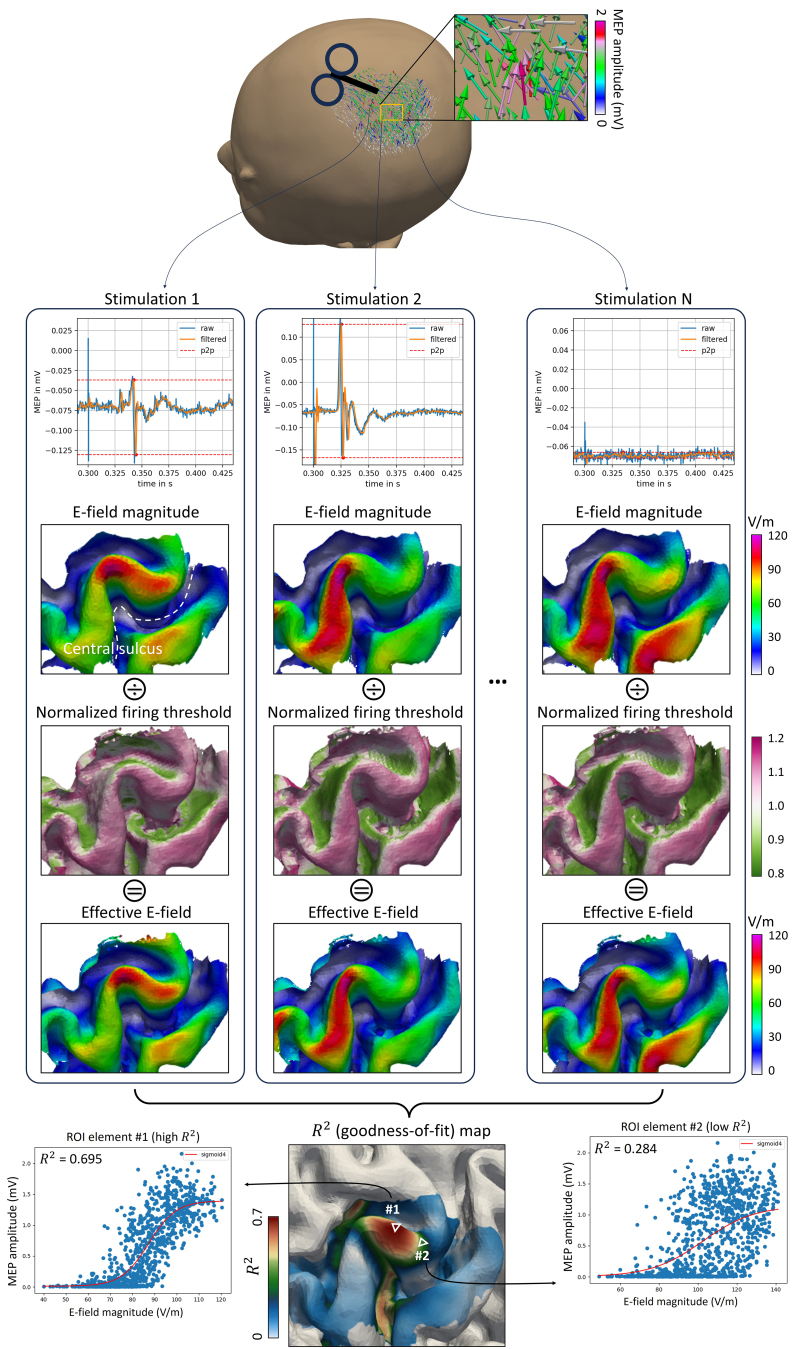
General principle of the regression-based TMS localization method. Top: Single-pulse TMS was applied at random coil locations and orientations around the M1 area while recording MEPs from the target muscle. Middle: For each TMS pulse (1~N), the corresponding E-field distribution in the cortical motor region was simulated using individual head models. The E-field magnitude maps were then divided by the normalized firing threshold $S\left(\theta ,\Delta |\;\;\tilde{E}\;|\right)$ to generate the effective E-field map. Bottom: For each cortical element within the motor cortex ROI, a sigmoidal regression was performed to fit the E-field quantities (|E|, $E_{eff}$ , or $\left|E_{⊥}\right|$) on the MEPs. The resulting goodness-of-fit maps identify the cortical region most strongly associated with the recorded MEPs, that is, the estimated cortical origin of the motor response.

We applied a regression-based localization method with random coil placements to identify muscle representations in the motor cortex where the neural populations are responsible for the TMS-induced MEPs. The core concept of this method is illustrated in Figure 3. The rationale is that at the cortical origin of the MEPs, a clear sigmoidal relationship must exist between induced E-field and MEP amplitude. This sigmoidal or s-shaped curve, known as the input-output curve (IO curve), captures how targeted motor neurons respond to varying stimulation strengths. At low stimulation strength (low |E|), MEP amplitudes remain below a baseline or noise floor, which is likely due to unrelated neural and muscular sources. As the stimulation strength increases, MEP amplitudes rise monotonically until reaching saturation, forming the high-side plateau of the sigmoidal IO curve (Li et al., 2022). Thus, we identified the cortical origin by selecting the location with the highest goodness-of-fit (GOF) for the sigmoidal regression (Fig. 3).

We propose that the regression based on the *neuron model* is the most accurate approximation of the effects of TMS-induced E-fields on MEPs. This method correlates $E_{eff}$ at layer 2/3 and layer 5 pyramidal cells with the measured MEP amplitudes using a sigmoidal function. As the $E_{eff}$ accounts for axonal orientations and other neuronal morphologies, this method should describe the TMS-induced effects on brain activities in the most realistic manner.

Previously employed methods like the *magnitude model* and the *cosine model* can be viewed as approximations of the neuron model’s directional profile. To test these approaches against the neuron model, |E| and $\left|E_{⊥}\right|$ on the midlayer were also used to regress with MEPs. The magnitude model assumes that the magnitude of the E-field (| E | ) best predicts the MEPs, whereas the cosine model considers the normal component of the E-field toward the cortical surface normal ($E_{⊥}$) responsible for the resulting MEPs (Fig. 2B). Here, we assume that the same magnitude of $E_{⊥}$ with opposite direction alongside the somato-dendritic axis yields the same neural activation, and, accordingly, we utilize the absolute value of $E_{⊥}$ ($\left|E_{⊥}\right|$) in our calculations. To reduce cases where $E_{⊥}$ becomes negative, TMS coil was positioned within a ± 60° range relative to the PA 45° (posterior-anterior 45 degrees) orientation, which aligns well with the typical axonal orientation in motor cortical neurons. We hypothesize that the magnitude model provides a better approximation compared to the cosine model based on the greater similarity of its angular profile to that of the neuron model (Fig. 2D).

A sigmoidal IO curve was used to characterize the relationship between the three kinds of E-field quantities (Fig. 2D) with the elicited MEPs for every element within the ROI, respectively:

$${\hat{y}}_{i,j}=\;y_{0}+\;\frac{a_{j}-\;y_{0}}{1+\;e^{-r_{j}(x_{i,j}-\;x_{0,j})}}$$

Here, $x_{i,j}$ is the E-field of TMS pulse i ($1\leq i\leq N_{TMS}$ ) at cortical element *j* ($1\leq j\leq N_{elms}$ ), and ${\hat{y}}_{i,j}$ is the fitted MEP, a defines the saturation amplitude, r is the slope, $x_{0}$ is the location of the turning point on the x-axis, and $y_{0}$ denotes the floor value that corresponds to measurement noise.

The site of effective stimulation can be quantified by the GOF, which is highest at the cortical site that houses the relevant neuronal populations (Fig. 3). We assessed the element-wise GOF by the coefficient of determination $R^{2}$:

$$R_{j}^{2}=1-\;\frac{VAR\;(y-\;{\hat{y}}_{j})}{VAR\;\left(y\right)}$$

Here, y is the measured MEP, and $\hat{y}$ is the fitted MEP. Higher $R^{2}$ denotes better fitting results.

We then compared the $R^{2}$ results obtained from the $E_{eff}$ at L2/3 and L5 with those derived solely from the |E| and $\left|E_{⊥}\right|$ by the Friedman test, a non-parametric repeated measures ANOVA. Post hoc pairwise comparisons were performed using Wilcoxon signed-rank tests with Holm correction. Due to the spatial autocorrelation (or “smoothness”) of the E-field, neighboring deep areas may also exhibit spurious correlations between E-fields and MEP amplitudes. To account for this, we limited $R^{2}$ peaks to regions where at least 25% of TMS trials had |E| exceeding 40 V/m. Additionally, we restricted $R^{2}$ peaks to the precentral gyrus as it houses the primary motor cortex, responsible for motor control. Limiting the analysis to this region ensures that the observed $R^{2}$ peaks are directly relevant to motor function and TMS stimulation effects.

#### 2.4.5 Convergence analysis

We conducted a convergence analysis on these three models using Dataset 1 to evaluate their stability and efficiency by determining the minimum number of TMS pulses needed for a robust cortical localization, following the method of Numssen et al. (2021). For each subject, we first randomized the sequence of all TMS pulses. Sequential regressions were then computed for n = 10 to 800 pulses to quantify the pulse-by-pulse change of the resulting $R^{2}$ map. This process was repeated 100 times with random initializations to ensure that $R^{2}$ maps converged to a stable solution regardless of the initial parameter values. The analysis began at n = 10 and ended at 800, based on previous findings (Numssen et al., 2021), which indicate that localization results do not converge before the 10th pulse and generally stabilize around the 200th pulse. This procedure resulted in 79100 $R^{2}$ maps (100 random sequences × 791 TMS pulses).

These $R^{2}$ maps were then used to compute two metrics: 1) the normalized root mean square deviation (NRMSD) to measure the overall shape similarity of the cortical localization $R^{2}$ map; and 2) the geodesic distance of $R^{2}$ peaks to quantify the accuracy of hotspot identification. Convergence for both metrics was evaluated against the full set of TMS pulses N as a proxy for the ground truth, as well as against the previous solution from n−1 pulse, to measure the speed of convergence.

The NRMSD between the $R_{n}^{2}$ map for n pulses and the reference map $R_{ref}^{2}$ was calculated as follows:

$$NRMSD=\;\frac{\sqrt{\frac{1}{N_{elms}}\;\sum_{i=1}^{N_{elms}}{\left(R_{i,n}^{2}-R_{i,ref}^{2}\right)}^{2}\;}}{max\;\left(R_{ref}^{2}\right)-min\;\left(R_{ref}^{2}\right)\;}$$

Here, i denotes the elements ($1\leq i\leq N_{elms}$ ) in the motor ROI, and the reference map $R_{ref}^{2}$ is either the $R^{2}$ at the full set of pulses N or at n−1 pulses.

We set convergence thresholds at 5% for NRMSD and 1 mm for the geodesic distance between hotspots. The minimum number of TMS pulses needed to meet these criteria against the reference solution is determined as the threshold for achieving stable cortical localizations. This convergence analysis provides both a practical stopping criterion for online TMS experiments and a method for post-hoc evaluation of the overall effectiveness of the mapping procedure’s effectiveness. To obtain robust convergence estimates for each subject, we averaged the minimum number of TMS pulses meeting the convergence criteria across 100 randomized sequences for each pulse count (from 10 to 800).

To determine which model yields the most stable cortical localization ($R^{2}$ map), we compared the mean minimum number of TMS pulses required to meet the NRMSD and geodesic distance thresholds across the three models at the group level using the Friedman test. Conover’s Post-hoc analysis was then conducted to identify significant differences between the models, with multiple-comparison corrections for pairwise comparisons. For the neuron model, we only used data from L5, as it produced similar localization results to those from L2/3.

### 2.5 TMS validation

Participants in Dataset 2 took part in both the localization and validation sessions. Following the TMS localization, in which the neuronal populations responsible for TMS-induced MEPs were identified based on three neuronal response models, we conducted a second session to validate these results on the 10 subjects in Dataset 2.

#### 2.5.1 Coil optimization

For each subject and each of the three models, we determined the optimal coil placement, that is, coil position and orientation, that yielded the maximum $E_{eff}$ , |E|, or $\left|E_{⊥}\right|$ at the $R^{2}$ peak, respectively. This was done using an exhausting search procedure, which involved projecting the hotspots onto the head surface and defining search grids centered on these projected sites with a radius of 20 mm, angle range of 360°, spatial resolution of 2 mm, and angle resolution of 4°, resulting in 27450 coil configurations for each model (Fig. 7A). E-field distributions were then simulated within the motor ROI for each coil configuration. The coil placements that yield the maximum $E_{eff}$ from L5 (for the neuron model), |E| (for the magnitude model), and $\left|E_{⊥}\right|$ (for the cosine model) at the corresponding $R^{2}$ hotspots were imported to the neuronavigation system (Kalloch & Numssen, 2022). This optimization procedure is implemented in SimNIBS (https://simnibs.github.io/simnibs).

In addition to the three model-based optimal coil placements (magnitude, cosine, and neuron), we included four surrounding control coil placements. These shared the same optimal coil orientation as the magnitude-model-based coil orientation but were shifted 10 mm in the superior, posterior, inferior, or anterior directions relative to the original magnitude coil placement (Fig. 7B). In total, seven coil placements were tested. To minimize participant burden and avoid excessive stimulation, we did not test additional coil orientations. This decision was supported by previous studies demonstrating that the optimal orientation reliably produces the lowest rMT (Numssen et al., 2021; Weise et al., 2020).

#### 2.5.2 Validation experiment

To validate the mapping results, we applied TMS at each of the seven coil placements using the same protocol as outlined in the localization experiment (see Section 2.4.1), with single biphasic pulses at a 5-s ISI. The stimulation intensity was set to 120% rMT instead of 150%, as this level typically yields robust MEPs while remaining highly sensitive to minor intensity changes (Houdayer et al., 2008; Souza et al., 2022). For each coil placement, 50 TMS pulses were collected to obtain a stable MEP estimate from the FDI muscle (Goldsworthy et al., 2016). The order of coil placements was randomized per subject to mitigate sequence effects.

MEP amplitudes across seven coil placements (three model-based and four control placements) were analyzed using a linear fixed-effects model (LMM) with *Coil Placement* as a fixed factor (seven levels: magnitude, neuron, cosine, anterior, posterior, superior, inferior) and *Subject* as a random intercept to account for within-subject variability while preserving all trial-level information:

log(MEP) ~ CoilPlacement + (1|Subject)

Because MEP amplitudes are positive-skewed, we applied a log transformation to reduce variance and improve normality. Post-hoc pairwise comparisons (Holm-corrected) were performed to test whether each model-based coil placement elicited higher MEP amplitudes than the four control sites, and to compare performance among the three model-based placements.

We hypothesized that the coil placements derived from the magnitude and neuron models would elicit higher MEP amplitudes than those derived from neuron model and nearby control placements, as these models more accurately locate the neuronal populations for MEPs. Here, we used the MEP amplitude as the index of corticospinal excitability instead of rMT because it provides a continuous, high-resolution measure of local motor output, allowing sensitive comparisons between coil placements.

## 3 Results

### 3.1 Localization maps

**Fig. 4.**
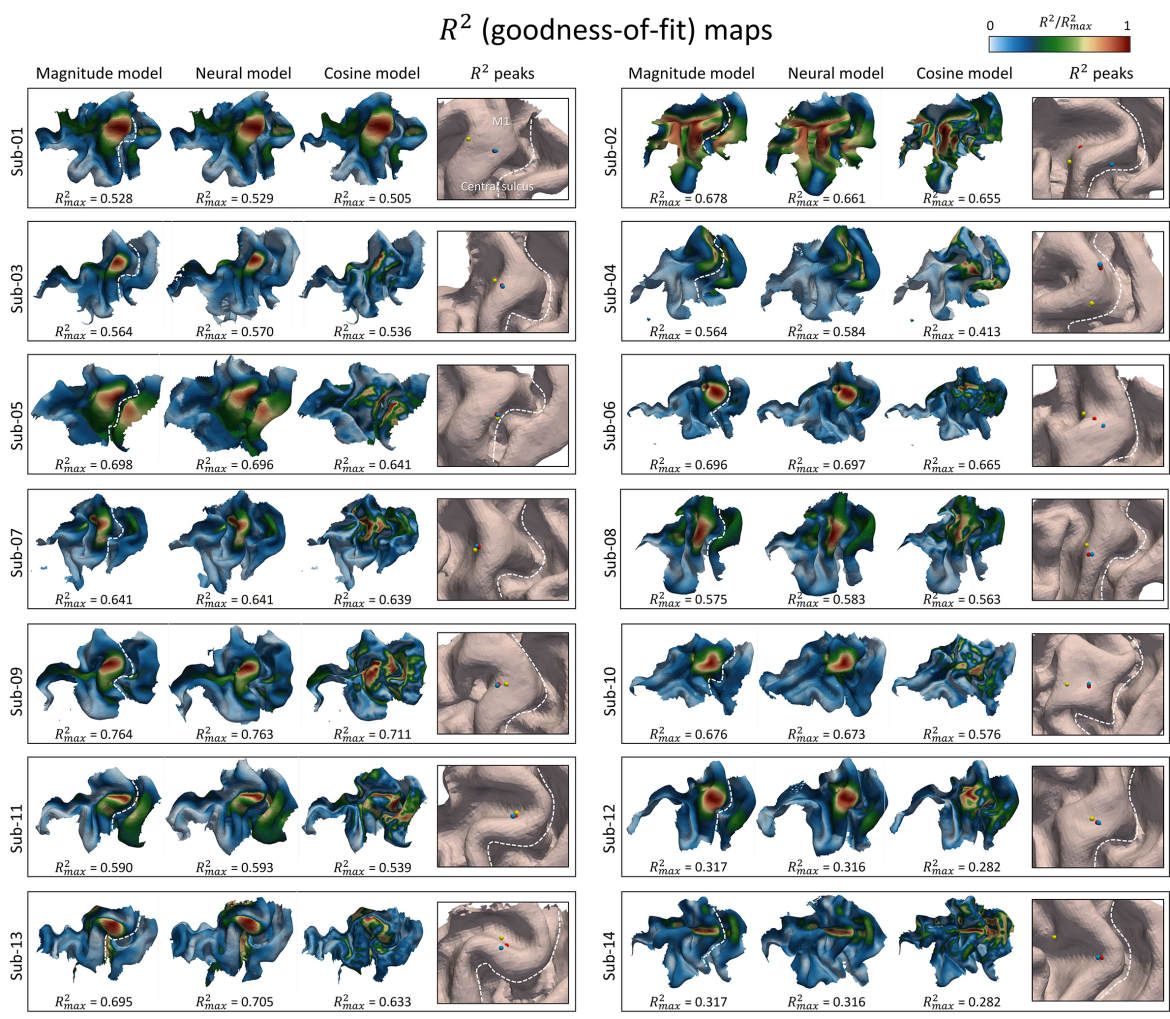
Individual motor mapping results for magnitude, neuron, and cosine response models. First three columns: normalized $R^{2}$ (goodness-of-fit) maps from magnitude model (|E|) on gray matter midlayer, neuron model (E_eff_) in layer 5 (L5), and cosine model (|E_ ┴_|) on midlayer. Maximum $R^{2}$ values range from 0.28 to 0.77. The cosine model yields lower $R^{2}\;$ values compared to the magnitude and neuron model, thus showing poorer performance in localizing cortical muscle representations. The last column highlights the identified hotspot for all three models. The locations derived from the magnitude (blue) and neuron (red) models are similar, while the hotspot from the cosine model (yellow) is significantly distant.

We performed a sigmoidal regression between the three E-field quantities and TMS-elicited MEPs to localize the cortical sites responsible for the observed MEP in the right FDI muscle. The effective stimulation site is assumed to have the highest $R^{2}$ value at the cortical location housing the relevant neuronal populations. Subject-wise localization results from Dataset 1 for the magnitude, neuron, and cosine models are presented in Figure 4 and Supplementary Table S1, while results from Dataset 2 are shown in Supplementary Figure S1 and Supplementary Table S3. Given the smaller sample size in Dataset 2, we focus on Dataset 1 in the main text, with Dataset 2 results detailed in the supplementary materials.

A Friedman test revealed a significant difference in $R^{2}$ peak values across the three neuronal response models (χ² = 23.229, *p* < 0.0001). We then performed Post hoc pairwise Wilcoxon signed-rank tests with Holm correction to identify significant pairwise differences.

The overall shapes of the normalized $R^{2}$ maps are very similar between the magnitude and neuron models, which incorporated neuron morphologies from L2/3 and L5. Their $R^{2}$ peak values are also not significantly different (mean_mag_ = 0.598; mean_neuron_L2/3_ = 0.600; mean_neuron_L5_ = 0.600; magnitude vs. neuron (L2/3): *Z* = 43.5, *p* = 0.58; magnitude vs. neuron (L5): *Z* = 49.5, *p* = 0.90). Given the similarity in results between L2/3 and L5, we focused on the L5 data for subsequent analyses.

**Fig. 5.**
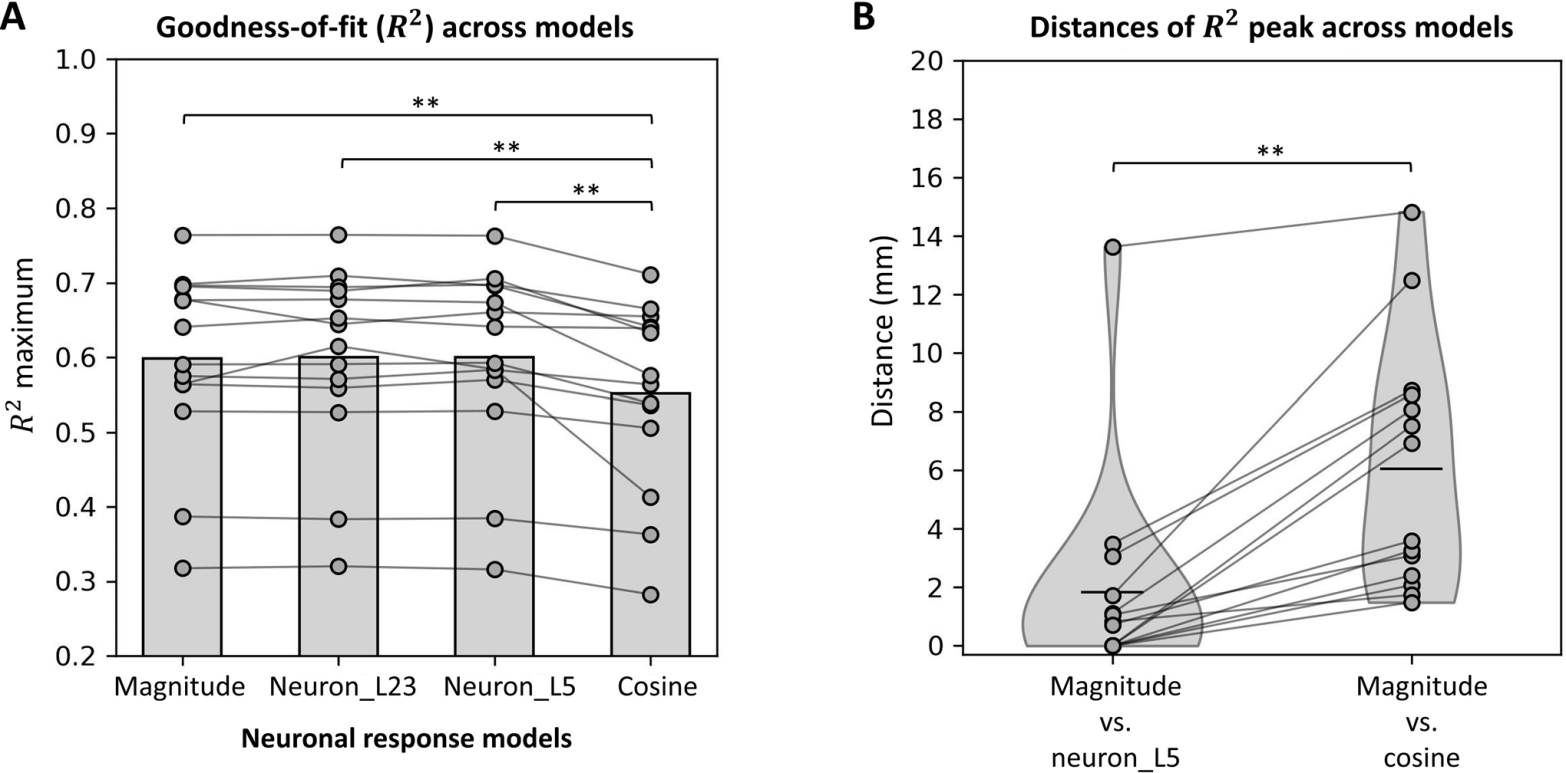
Comparisons of $R^{2}$ (goodness-of-fit) peak values and locations across neuronal response models. (A) The cosine model exhibits the lowest $R^{2}$ peak values, indicating that the normal component of electric fields (E-fields) explains less variance of motor-evoked potentials (MEPs) compared to other models. (B) Hotspot locations identified by the magnitude model are significantly closer to those identified by the neuron model than with the cosine model. Black lines represent mean distances between models. **p < 0.01.

In contrast, the cosine model shows significantly lower $R^{2}$ peak values compared to the magnitude model (mean_cos_ = 0.551; *Z* = 0.0, *p* < 0.001). A test statistic of *Z* = 0.0 indicates that for all data pairs, the $R^{2}$ peak values from the cosine model are consistently lower than those from the magnitude model. Similarly, the comparison between the cosine and neuron models also shows a significant difference (*Z* = 0.0, *p* < 0.001), further confirming that the cosine model provides a less accurate functional localization of the cortical origin of the elicited MEPs. Moreover, the $R^{2}$ maps from the cosine model appear noisier than those from the magnitude and neuron models, indicating reduced spatial coherence and accuracy in cortical localization. Figure 5A and Supplementary Table S1 provides both individual and group mean $R^{2}$ peak values for all models across all subjects in Dataset 1.

Likewise, the locations of maximum $R^{2}$ values are significantly closer between the magnitude and neuron model compared to the distance between the magnitude and cosine models (Fig. 5B, Supplementary Table S2, *Z* = 0.0, *p* < 0.0001). This highlights the strong agreement between the magnitude and neuron models in identifying cortical hotspots, while the cosine model shows more divergence.

### 3.2 Convergence analysis

**Fig. 6.**
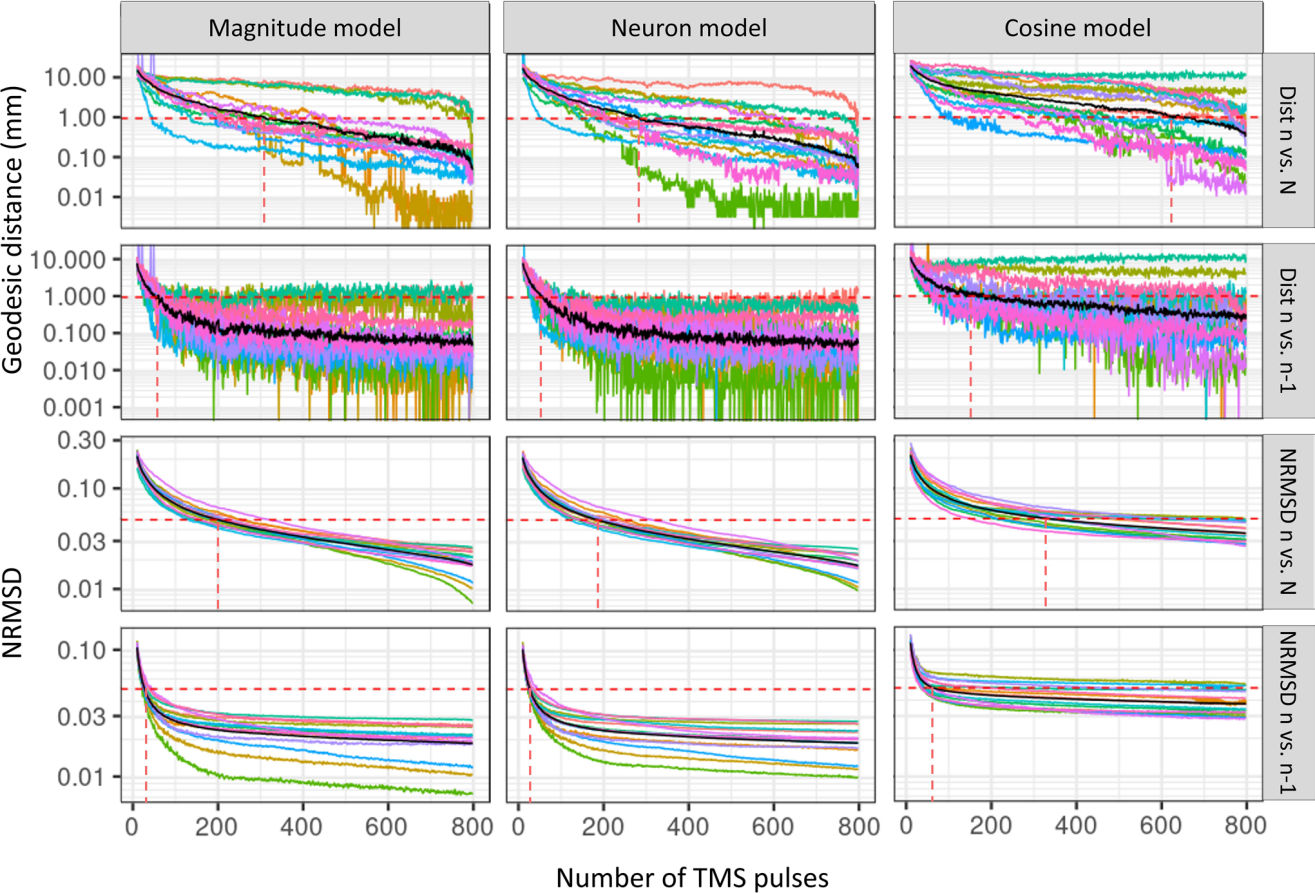
Comparison of motor localization ($R^{2}$ map) convergence across models. The magnitude and neuron models converge faster than the cosine model, i.e., they require fewer TMS pulses to achieve stable mapping results. The first two rows show the geodesic distance of the identified hotspot from the current n stimulations compared to both the reference solution (N, the full dataset) and the previous solution (n−1 TMS pulses). The last two rows show normalized root mean square deviation (NRMSD), which quantifies map similarity between the maps from n TMS pulses and both the reference solution (N) and the previous solution (n−1 ). Colored lines: subject-wise average convergence across 100 randomizations. Black lines: the grand mean. Red dashed line: the number of TMS pulses required to reach a 1 mm geodesic distance and 5% NRMSD relative to the reference solution N and previous solution n−1 , respectively. Convergence plots were cut at 800 pulses. Dist: geodesic distance.

We conducted a convergence analysis to determine which model requires fewer TMS pulses to achieve a stable and correct cortical localization, quantified as the overall similarity of $R^{2}$ maps (NRMSD) and geodesic distance of $R^{2}$ peak locations (Fig. 6). Both metrics were used to compare each model’s n-th solution to both the ground truth (N TMS pulses) and the previous solution (n−1 TMS pulses). Overall, both the magnitude and neuron models converged faster than the cosine model.

For the geodesic distance between $R^{2}$ peak locations from the n-th solution to the ground truth, the magnitude model required 309 TMS pulses, the neuron model required 282 TMS pulses, and the cosine model required 622 TMS pulses to reach the 1 mm threshold. A Friedman test revealed a marginal difference among these three models in terms of the minimum number of TMS pulses needed for stable $R^{2}$ peak locations ($\chi^{2}$ = 5.57, *p* = 0.06). Conover’s post hoc test, corrected for Bonferroni multiple comparisons, further indicated that the cosine model required significantly more TMS pulses to reach stability compared to both the magnitude model (*Z* = 2.25, *p* = 0.045) and neuron models (*Z* = 2.57, *p* = 0.02). No significant difference was found between the magnitude and neuron models (*Z* = -0.32, *p* = 1.00).

When comparing the current solution n with the previous n−1 solution, the magnitude model required 59 TMS pulses, the neuron model 57 TMS pulses, and the cosine model 152 TMS pulses to achieve stable peak distances. A Friedman test showed a significant difference across models ($\chi^{2}$ = 18.58, *p* < 0.0001). Post hoc analysis confirmed that the cosine model needed significantly more pulses to reach continuous localization results than both the magnitude (*Z* = 4.79, *p* < 0.0001) and neuron models (*Z* = 4.89, *p* < 0.0001). No significant difference was found between the magnitude and neuron models (*Z* = -0.10, *p* = 1.00).

In terms of NRMSD, which quantifies the overall similarity of two $R^{2}$ maps, the magnitude and neuron models required 200 and 191 TMS pulses, respectively, to reach the 5% deviation threshold compared to the full set of N pulses. The cosine model, however, required 278 TMS pulses to reach the same threshold. A Friedman test revealed a significant difference across models ($\chi^{2}$ = 24.14, *p* < 0.0001), and post hoc analysis confirmed that the cosine model converged significantly slower than both the magnitude (*Z* = 3.92, *p* < 0.0001) and neuron models (*Z* = 4.83, *p* = 0.0005). Additionally, 31 and 28 TMS pulses were needed for the magnitude and neuron models to produce a similar localization pattern compared to the previous solutions, while the cosine model required 61 TMS pulses. A Friedman test showed a significant difference across models ($\chi^{2}$ = 25.08, *p* < 0.0001), with post hoc analysis indicating that the cosine model again converged significantly slower than both the magnitude (*Z* = 5.75, *p* < 0.0001) and neuron models (*Z* = 6.82, *p* < 0.0001).

These findings highlight that both the magnitude and neuron models converge more quickly and require fewer TMS pulses to achieve stable and continuous localization patterns, while the cosine model lags behind, requiring significantly more pulses to reach the same level of stability. This suggests that the magnitude and neuron models provide more efficient and reliable cortical localization compared to the cosine model.

### 3.3 Validation

**Fig. 7.**
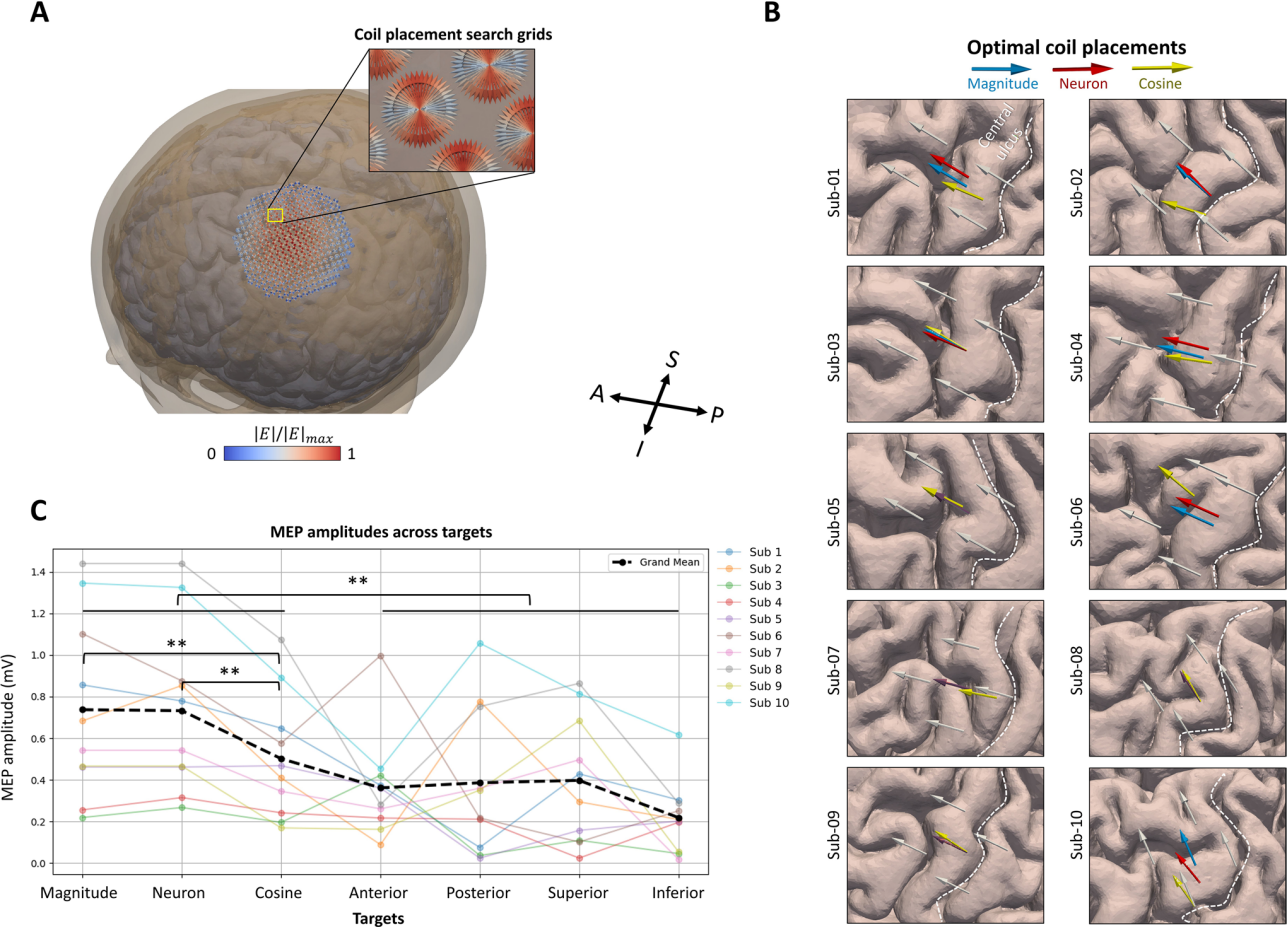
Experimental validation of the identified muscle representations across the three activation models. (A) An exhaustive search procedure was performed, testing 27450 coil placements (arrows) for each model to find the coil placement that maximizes the E-field quantity at the corresponding $R^{2}$ peak hotpot. (B) Optimal coil placements for the three models, along with four surrounding control sites at the individual level. The tail of each arrow indicates the coil location, while the arrow’s direction represents coil orientation. Purple arrow: the overlap between coil placements from magnitude (blue) and neuron (red) models at layer 5 (L5). (C) The magnitude and neuron models yielded higher motor-evoked potential (MEP) peak-to-peak amplitudes compared to the cosine model and the four surrounding control sites, implying better localization of the cortical muscle representations. Colored points: subject-wise mean MEP amplitudes for 50 TMS pulses. Black dashed lines: the grand mean. A, anterior; P, posterior; S, superior; I, inferior. **p < 0.01.

To validate the cortical mapping results from the three neuronal response models, we calculated the optimal coil placements on Dataset 2 for the identified cortical hotspots (Supplementary Table S4). Experiments were then conducted with the guidance of these calculated optimal coil placements, and MEP amplitudes were measured to evaluate the accuracy and reliability of each model’s mapping. In addition to the three optimized coil positions derived from the magnitude, neuron, and cosine models, four adjacent coil placements were tested as control conditions to provide comparative data (Fig. 7C, Supplementary Table S5).

The LMM revealed a significant main effect of coil placement on log-transformed MEP amplitudes (likelihood ratio test: $\chi_{\;\;\;\;(6)}^{2}=609.13$ , *p* < 0.001), indicating that stimulation site significantly influenced corticospinal excitability. Estimated marginal means (back-transformed to the original scale, ± SE) showed that MEP amplitudes were highest for the magnitude (0.45 ± 0.09) and neuron (0.44 ± 0.09) placements, followed by the cosine model (0.24 ± 0.05), and were lowest at the control sites (anterior: 0.15 ± 0.03; posterior: 0.12 ± 0.03; inferior: 0.07 ± 0.02; superior: 0.16 ± 0.03).

Post hoc pairwise comparisons (Holm-corrected) indicated that both the magnitude and neuron coil placements produced significantly higher MEP amplitudes than all four control sites (all *p* < 0.001, Cohen’s *d* = 0.73 – 1.28). Within the model-based placements, the magnitude and neuron models did not differ significantly (*t_(9)_* = 0.34, *p* = 0.74, *d* = 0.02), whereas both yielded significantly higher MEP amplitudes than the cosine model (magnitude vs. cosine: *t_(9)_* = 7.64, *p* < 0.001, *d* = 0.49; neuron vs. cosine: *t_(9)_* = 7.27, *p* < 0.001, *d* = 0.47).

The cosine model also produced significantly higher MEP amplitudes than all control sites (all *p* ≤ 0.001), although with smaller effect sizes (*d* = 0.26 – 0.78), indicating weaker and less consistent performance compared to the magnitude and neuron models.

Post hoc power analysis confirmed that with sample size n = 10, our design could detect effects as small as *d* = 0.93 with 80% power, confirming that the sample size provided sufficient sensitivity to detect robust within-subject effects.

Together, these findings suggest that the magnitude and neuron models more accurately identified the cortical areas responsible for generating MEPs in the target hand muscle, outperforming the cosine model and nearby control sites. Notable, four subjects even exhibited identical optimized coil placements for the magnitude and neuron models (Fig. 7B).

## 4 Discussion

In TMS mapping, the conventional approach predominantly relies on the magnitude of the induced E-field (|E|) to approximate neuronal activation and excitability (Hikita et al., 2023; Jing et al., 2023; Numssen et al., 2021; Weise et al., 2020). However, axons are activated by differences in potential along their length, rendering directional sensitivity as a potentially crucial factor besides the E-field strength (Numssen et al., 2024; Ranck Jr, 1975; Rushton, 1927). This led to the development of the cosine model, which assumes that axonal depolarization is determined primarily by the normal component of the E-field parallel to the axon ($\left|E_{⊥}\right|$) (Fox et al., 2004). Although this model introduces directional sensitivity, its assumption that neurons are predominantly oriented downward and perpendicular to the cortical surface as the sole point of TMS-induced activation may be overly strong and restrictive. While including macroscopic properties like gyrification patterns, both two activation models neglect mesoscopic details, such as the complex morphology of cortical neurons and their varied spatial organization within the cortical layers. This neuronal architecture might further complicate how the E-field interacts with neurons, influencing local cortical activation.

To provide a more physiologically informed approach, we incorporated the average response model into the regression-based TMS mapping framework. In our implementation, this approach is referred to as the *neuron model*, which uses the effective E-field **($E_{eff}$ )** as the E-field quantity to estimate neuronal responses to stimulation. The effective E-field scales the E-field magnitude |E| based on neuron-specific firing thresholds, providing a more refined understanding of how TMS-induced E-fields influence motor responses (i.e., MEPs). Rather than replacing the widely used magnitude model, our approach serves as a test rig for evaluating different neuronal response models (i.e., magnitude and cosine models).

Our findings indicate that, in the motor cortex, E-field magnitude remains the primary driver of neuronal activation, while the normal component plays a relatively minor role. This suggests that, for practical motor mapping applications, the magnitude model remains a highly effective and computationally efficient choice. However, our results also highlight the need for more detailed, layer-specific data that incorporate both macroscopic and microscopic cortical features in future modeling efforts.

### 4.1 Cortical localization: magnitude and neuron models outperform the cosine model

Our regression-based mapping approach differs from conventional motor hotspot-hunting methods that locate the optimal scalp position for eliciting the smallest rMT or largest MEP amplitude (Harquel et al., 2017; Meincke et al., 2016; Tervo et al., 2020). Rather than focusing on coil optimization at the scalp level, our method estimates the cortical origin of MEP by modeling how the neuronal populations respond to TMS-induced E-fields within the cortex. We performed a sigmoidal regression analysis to localize the cortical representation of the target muscle and compared three neuronal response models, i.e., the magnitude, neuron, and cosine models, to examine how different E-field quantities affect the precision of cortical localization.

The *magnitude* and *neuron models* (incorporating neuron morphologies from cortical L2/3 and L5) demonstrated similar localization results, both in terms of the overall shape of the $R^{2}$ maps, the $R^{2}$ peak values, and the locations of $R^{2}$ peaks. This indicates that accounting for neuron-specific factors derived from an average response model does not significantly alter the overall localization compared to using the magnitude of the E-field alone. In other words, the magnitude model alone is already an effective, simple, and fast approach in identifying the relevant cortical sites responsible for the MEPs. Results showed that the FDI muscle representation is primarily located on the rim and the crown of the precentral gyrus, in accordance with previous studies (Aonuma et al., 2018; Bungert et al., 2017).

In contrast, the *cosine model*, which assumes that stimulated axonal segments are exclusively oriented orthogonally to the cortical surface, explained significantly less variance in the regression compared to both the magnitude and neuron models. The consistently lower $R^{2}$ values across all subjects indicate that the cosine model is less effective at capturing the functional stimulation site compared to the other two models. Moreover, $R^{2}$ maps generated by the cosine model lacked the spatial coherence seen in the magnitude and neuron models, indicating more noise was introduced into the localization process. The distinct locations of maximum $R^{2}$ values between cosine and the other two models further confirmed this. Accordingly, we do not recommend the cosine model to be used in future mapping studies.

In fact, the E-field strength is generally higher in the gyral crown, which is closer to the scalp, while the normal component of the E-field (as captured by the cosine model) is more prominent in the sulcal wall. This observation aligns with our results, showing distinct localization pattern differences between the cosine model and the other two models. Notably, functional MRI studies have found that TMS activates deeper cortical regions, such as the sulci, primarily due to proprioceptive sensory feedback from muscle twitches. When this feedback was suppressed in concurrent TMS-fMRI studies, however, the activation shifted closer to the gyral crown, areas of higher E-field magnitude rather than the normal component emphasized by the cosine model (Shitara et al., 2013). This supports our conclusion that compared to the cosine model, the magnitude model is better suited for mapping the cortical sites responsible for MEPs.

### 4.2 Convergence analysis: magnitude and neuron models demonstrate faster convergence

The convergence analysis revealed significant differences in how quickly each model achieves stable cortical localization with respect to the number of TMS pulses. Both the magnitude and neuron models demonstrated faster convergence compared to the cosine model, as indicated by the geodesic distance of $R^{2}$ peak locations and the overall similarity $R^{2}$ maps measured by NRMSD.

In terms of the geodesic distance compared to the ground truth (N TMS pulses), which evaluates the precision of peak localization, the magnitude and neuron models required far fewer TMS pulses (309 and 282, respectively) to reach a 1 mm threshold compared to the cosine model, which needed 622 TMS pulses. However, no significant difference was found between the magnitude and neuron models, suggesting that neuron-specific adjustments do not drastically improve convergence speed over the magnitude model alone. Similarly, the NRMSD analysis, which assesses the overall similarity between $R^{2}$ maps, further supported the superiority of the magnitude and neuron models. Both required fewer than 200 TMS pulses to reach a 5% deviation threshold, while the cosine model needed 278 TMS pulses to achieve the same stability.

In summary, these results emphasize that both the magnitude and neuron models outperform the cosine model in terms of convergence speed and efficiency, making them better choices for future TMS studies. Given they converged at similar rates, the magnitude model stands out as a simpler and faster approach, offering efficient localization without the need for complex neuron-specific adjustments. We recommend at least 300 TMS pulses are needed for the regression-based TMS mapping to get stable localization results.

### 4.3 Validation: magnitude and neuron models yield better coil placements

The validation experiment in our study aimed to determine which model most accurately locates the cortical hotspots responsible for eliciting MEPS in the FDI hand muscle. The results demonstrated that both the magnitude and neuron models produced significantly higher peak-to-peak MEP amplitudes compared to the cosine model and the adjacent control positions. Moreover, 4 subjects even exhibited identical optimized coil positions for the magnitude and neuron models, further reinforcing their similarities in cortical localization. These findings validate the superiority of the magnitude and neuron models over the cosine model in pinpointing the cortical regions involved in generating MEPs. The lower MEP amplitudes observed in the cosine model and control positions suggest that these locations are less functionally relevant for eliciting strong MEP responses.

It is important to note that we did not test coil orientations beyond the calculated optimal coil orientation, which is typically around PA 45°. This orientation, where the TMS coil is positioned at a 45° angle to the mid-sagittal line over the primary motor cortex, generating a posterior-to-anterior current, is widely considered optimal for motor cortical stimulation (Brasil-Neto et al., 1992). One reason for this choice is that a previous study by Numssen et al. (2021) systematically examined alternative stimulation directions beyond PA 45° and confirmed that the optimal coil orientation consistently produced the highest MEP amplitudes. Given the need to minimize participant burden and avoid excessive stimulation, we did not include additional orientations beyond PA 45° in our study. Additionally, as shown in Supplementary Figure S2, the E-field is approximately strongest at this commonly used PA 45° orientation, reinforcing our decision to focus on this orientation.

### 4.4 Comparison among three neuronal response models

The comparable performance of the neuron and magnitude models suggests that, although the neuron model incorporates the directional sensitivity of cortical neurons, the additional complexity does not improve motor mapping accuracy under typical stimulation conditions. This likely reflects that, in the primary motor cortex, neuronal populations with varying orientations are activated simultaneously within a relatively broad E-field distribution, causing directional effects to average out at the spatial scale captured by current mapping resolutions. Consequently, the simpler magnitude model already captures the dominant determinants of excitability. Although the neuron model provides a more biophysically informed approximation of neuronal activation, the impact of directional sensitivity appears moderate in the motor cortex and is limited to low stimulation intensities (Souza et al., 2022). As a result, both models yield similar localization outcomes.

In contrast, the cosine model underperforms because it oversimplifies neuronal directional sensitivity, assuming that all axons are oriented perpendicular to the cortical surface. Further, it assumes that neuronal activation is dictated solely by the normal component of the E-field ($\left|E_{⊥}\right|$). This assumption is overly restrictive, as it does not accurately capture the complex orientations and morphologies of neurons in the motor cortex, particularly in deeper cortical layers (L2/3 and L5). Additionally, the cosine model’s emphasis on $\left|E_{⊥}\right|$ results in an overrepresentation of sulcal activations, which is inconsistent with empirical findings that show TMS preferentially stimulates the gyral crown, where |E| is strongest.

Thus, for practical applications, the magnitude model remains a computationally efficient and robust choice for TMS mapping. The neuron model serves as a biophysically informed model for validating neuronal response models, but does not drastically change localization outcomes. The cosine model is less suitable due to its restrictive assumptions. Future work should explore whether these findings extend to other cortical regions where directional sensitivity might play a larger role.

### 4.5 Limitations and future directions

A key limitation of this study lies in the use of computational neuron models derived from rodent data rather than direct human neuronal recordings. While we applied established methods to adapt Blue Brain cell models for better approximations of human-like neurons (Aberra et al., 2018; Weise, Worbs, et al., 2023), these adaptations may not fully capture all aspects of human neuronal excitability.

In addition, the average response model used to estimate neuronal excitability incorporates only a moderate degree of directional sensitivity. As illustrated in Figure 2D, when the polar angle θ increases from 0° to 90°, the cosine model predicts a steep drop to zero, suggesting that neurons would not be activated when the E-field is orthogonal to the axon. In contrast, the $E_{eff}$ , derived from the average response model, decreases more gradually and remains relatively close to the |E|. This moderate tuning implies that the neuron model may underestimate the suppressive effects when the E-field orientation deviates significantly from the axon. Future work should explore more refined neuronal models that more accurately capture the directional dependence of excitability.

Furthermore, the biophysical modeling of TMS-induced E-fields has inherent limitations that must be considered when interpreting our findings. Head model reconstruction is subject to segmentation errors, as the differentiation of brain tissues (e.g., gray matter, white matter, and cerebrospinal fluid) from MRI data depends on automated segmentation algorithms, which introduce small but systematic inaccuracies in tissue boundaries. These inaccuracies can affect the precise localization and intensity of simulated E-fields. In addition, the conductivity values used in FEM simulations are derived from literature estimates rather than individualized conductivity measurements, potentially leading to deviations from actual physiological conditions. Taken together, these factors likely introduce inaccuracies in E-field simulations and may partly explain the lack of significant improvement in neuronal activation predictions when using the neuron model compared to the standard magnitude model.

Finally, our experimental control design also carries spatial limitations. In the validation experiment, four control coil placements were displaced by 10 mm along the anterior–posterior and inferior–superior axes. This approach introduced two inherent limitations. First, because these control sites were defined in scalp coordinates, they were not always positioned over homologous cortical regions across subjects, making MEP amplitudes from these sites less comparable between subjects. Second, the fixed 10 mm spacing may have overlooked additional responsive sites at smaller spatial scales.

Future studies could extend the analysis of $R^{2}$ maps beyond identifying local maxima by examining additional spatial characteristics such as spatial extent, shape, and sigmoidal turning point ($x_{0}$). For example, a focal, unimodal $R^{2}$ distribution likely reflects a well-organized and specific motor representation. Whereas a more diffuse or irregular distribution, although partly driven by the spatial spread of the induced E-fields, could indicate reduced cortical specificity or compensatory recruitment of neighboring areas (Durner et al., 2024). However, since shape or extent information of the map is strongly overlaid by the spatial point-spread function reflecting correlations between the leadfields across locations, this will need further methodological development.

Similarly, the sigmoidal turning point ($x_{0}$) provides complementary information on local excitability, as lower $x_{0}$ values suggest that a region is more sensitive to stimulation and may reach activation thresholds more readily. These map characteristics could serve as complementary functional biomarkers for assessing motor function integrity and complexity (Morishita et al., 2025), particularly in the contexts of stroke recovery, healthy aging, and developmental plasticity (Säisänen et al., 2021).

## 5 Conclusions

In this study, we applied a regression-based localization method to identify the cortical representation of muscle responses to TMS. We focused on modeling the induced E-field across different coil placements to investigate whether its distribution could explain physiological observations related to MEPs. We evaluated three different models for cortical localization: the magnitude model (regardless of E-field direction), the neuron model (accounting for both E-field magnitude and directional sensitivity), and the cosine model (overemphasizing the directional sensitivity of neurons). This is the first study to attempt cortical mapping that integrates a realistic model of cortical neuron morphology and systematically compares results with traditional neuronal response models.

Our findings demonstrated that on the motor cortex, both the magnitude and neuron models consistently outperformed the cosine model in terms of precision, efficiency, and stability. Their faster convergence and higher accuracy in identifying cortical hotspots highlight their practical advantages for TMS studies. These findings imply that the simpler magnitude model is already highly effective and offers the added benefit of reduced computational demands, making it an accessible and robust choice for TMS mapping. The neuron model, however, represents a promising approach for integrating cortical neuron morphology, and may potentially enhance cortical mapping directly with respect to simulated cortical response functions, so long as detailed cell-layer data are available. When the cytoarchitectural details of a target region are unknown, our study suggests that the magnitude model could serve as a reasonable first approximation.

We, therefore, recommend the simpler magnitude model for future TMS mapping research in the motor cortex, requiring at least 300 TMS pulses for stable and reliable localization results. The cosine model, however, due to its slower convergence and lower precision, is not recommended for future studies.

## Supplementary Material

Supplementary Material

## Data Availability

The data are available on the Open Science Framework at: https://osf.io/9f3bc. The code is available on GitLab at: https://gitlab.gwdg.de/tms-localization/papers/neuron-regression.git.
