# Supplementary material for: Effects of electric field direction on TMS-based motor cortex mapping
Source: Imaging Neurosci (Camb). 2026 Apr 21;4:IMAG.a.1211. doi: 10.1162/IMAG.a.1211 (PMC13100673; doi:10.1162/IMAG.a.1211)
Supplement: Supplementary Material [file IMAG.a.1211_supp.pdf]

# Effects of Electric Field Direction on TMS-based Motor Cortex Mapping

# Supplementary Materials

## Subject-wise localization results from Dataset 2

A Friedman test revealed a significant difference in $R^2$ peak values across the three neuronal response models ($\chi^2$ = 21.143, $p$ < 0.0001). We then performed Post hoc pairwise Wilcoxon signed-rank tests with Holm correction to identify significant pairwise differences.

The normalized $R^2$ maps from Dataset 2 similarly demonstrate strong alignment between the magnitude and neuron models (Fig. S1). Their $R^2$ peak values are not significantly different ($mean_{mag}$ = 0.564; $mean_{neuron\_L5}$ = 0.565; magnitude vs. neuron (L5): $Z$ = 20.0, $p$ = 0.49).

In contrast, the cosine model showed a near-significant trend of lower $R^2$ peak values compared to both the magnitude ($mean_{cos}$ = 0.518; $Z$ = 10.0, $p$ = 0.08) and neuron models ($Z$ = 11.0, $p$ = 0.11). Given the small sample size (n = 10), it is possible that the lack of statistical significance is due to limited power rather than the absence of a true effect. Table S3 provides both individual and group mean $R^2$ peak values for all models across all subjects in Dataset 2.

The locations of maximum $R^2$ values were significantly closer between the magnitude and neuron models compared to the distance between the magnitude and cosine models (Table S4, $Z$ = 1.0, $p$ = < 0.001). This finding further highlights the strong agreement between the magnitude and neuron models in identifying cortical hotspots, whereas the cosine model shows more divergence.

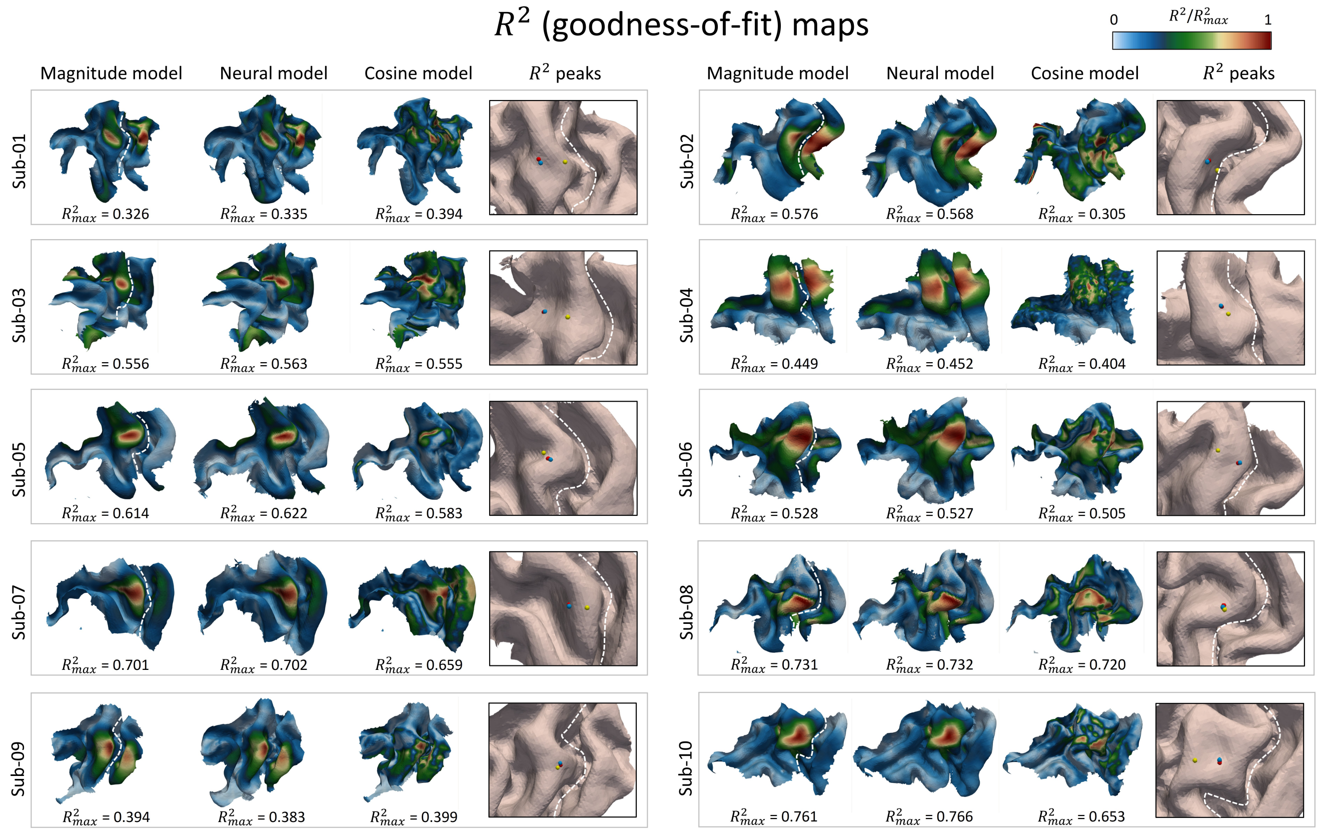


**Figure S1.** Individual motor mapping results for magnitude, neuron, and cosine response models from **Dataset 2**. First three columns: normalized $R^2$ maps from magnitude model ($|E|$) on gray matter midlayer, neuron model ($E_{eff}$) in L5, and cosine model ($|E_{\perp}|$) on midlayer. The last column highlights the identified hotspot for all three models.

**Table S1.** Subject-wise $R^2$ maximum values from three activation models

| $R^2$ maximum values | | | |
|---|---|---|---|
| Sub No. | Magnitude | Neuron | Cosine |
| 01 | 0.5279 | 0.5285 | 0.5053 |
| 02 | 0.6777 | 0.6605 | 0.6547 |
| 03 | 0.5638 | 0.5697 | 0.5361 |
| 04 | 0.5640 | 0.5838 | 0.4129 |
| 05 | 0.6979 | 0.6959 | 0.6409 |
| 06 | 0.6964 | 0.6974 | 0.6646 |
| 07 | 0.6406 | 0.6410 | 0.6390 |
| 08 | 0.5747 | 0.5831 | 0.5633 |
| 09 | 0.7638 | 0.7630 | 0.7111 |
| 10 | 0.6764 | 0.6734 | 0.5759 |
| 11 | 0.5903 | 0.5926 | 0.5386 |
| 12 | 0.3174 | 0.3158 | 0.2824 |
| 13 | 0.6949 | 0.7053 | 0.6329 |
| 14 | 0.3868 | 0.3845 | 0.3628 |
| Mean | 0.5980 | 0.5996 | 0.5515 |

***Note:*** The table presents subject-wise $R^2$ maximum values obtained from three activation models (Magnitude, Neuron, and Cosine). The data corresponds to **Dataset 1**. The cosine model exhibits the lowest $R^2$ peak values compared to other models.

**Table S2.** Subject-wise $R^2$ maximum coordinates from three activation models

| **$R^2$ maximum coordinates (x, y, z)** | | | |
|---|---|---|---|
| Sub No. | Magnitude | Neuron | Cosine |
| 01 | -28.12, -5.36, 64.13 | -27.87, -5.143, 63.63 | -24.58, -1.25, 66.35 |
| 02 | -34.27, -28.03, 65.81 | -25.97, -26.56, 64.99 | -26.12, -23.10, 63.36 |
| 03 | -35.93, -16.53, 74.76 | -35.31, -16.61, 74.36 | -30.59, -16.84, 70.42 |
| 04 | -31.63, -32.17, 49.80 | -31.77, -31.97, 49.07 | -36.34, -27.80, 46.55 |
| 05 | -27.00, -33.14, 79.85 | -26.64, -32.96, 79.76 | -27.19, -33.09, 78.90 |
| 06 | -36.52, -2.61, 65.72 | -34.23, -1.76, 65.19 | -30.85, -1.05, 61.76 |
| 07 | -30.33, -27.93, 65.37 | -30.71, -28.29, 65.44 | -30.16, -27.50, 63.75 |
| 08 | -34.38, 13.20, 70.26 | -33.84, 13.60, 69.35 | -31.83, 13.19, 69.70 |
| 09 | -33.11, -29.98, 71.08 | -32.85, -30.40, 70.70 | -34.01, -31.51, 71.73 |
| 10 | -33.65, -29.22, 65.77 | -33.94, -29.14, 65.20 | -32.40, -24.51, 66.65 |
| 11 | -28.96, -28.46, 54.47 | -28.88, -28.99, 53.60 | -27.23, -29.93, 51.77 |
| 12 | -31.49, -34.92, 56.42 | -31.13, -34.78, 56.00 | -30.36, -33.96, 56.68 |
| 13 | -25.73, -36.60, 57.93 | -24.97, -38.04, 55.87 | -22.13, -37.96, 51.19 |
| 14 | -39.70, -44.62, 50.50 | -40.47, -45.23, 51.07 | -29.34, -39.61, 46.54 |

***Note:*** The table presents subject-wise $R^2$ maximum coordinates $(x, y, z)$ for three activation models (Magnitude, Neuron, and Cosine). Data corresponds to **Dataset 1**. Maximum locations identified by the magnitude model are significantly closer to neuron model than to cosine model.

**Table S3.** Subject-wise $R^2$ maximum values from three activation models

| $\boldsymbol{R^2}$ **maximum values** | | | |
|---|---|---|---|
| Sub No. | Magnitude | Neuron | Cosine |
| 01 | 0.3258 | 0.3349 | 0.3935 |
| 02 | 0.5761 | 0.5678 | 0.3053 |
| 03 | 0.5563 | 0.5628 | 0.5547 |
| 04 | 0.4487 | 0.4515 | 0.4042 |
| 05 | 0.6135 | 0.6220 | 0.5833 |
| 06 | 0.5279 | 0.5268 | 0.5053 |
| 07 | 0.7005 | 0.7015 | 0.6593 |
| 08 | 0.7311 | 0.7317 | 0.7199 |
| 09 | 0.3935 | 0.3827 | 0.3987 |
| 10 | 0.7612 | 0.7658 | 0.6530 |
| **Mean** | **0.5635** | **0.5648** | **0.5177** |

***Note:*** The table presents subject-wise $R^2$ maximum values obtained from three activation models (Magnitude, Neuron, and Cosine). The data corresponds to **Dataset 2**.

**Table S4.** Subject-wise $R^2$ maximum coordinates from three activation models

| $\boldsymbol{R^2}$ **maximum coordinates (x, y, z)** | | | |
|---|---|---|---|
| Sub No. | Magnitude | Neuron | Cosine |
| 01 | -35.31, -15.99, 43.36 | -34.36, -16.08, 43.14 | -37.57, -20.07, 41.37 |
| 02 | -43.90, -35.10, 39.36 | -43.03, -35.32, 38.40 | -42.44, -35.42, 33.62 |
| 03 | -23.95, -30.45, 71.97 | -23.79, -30.66, 71.27 | -28.91, -27.91, 80.20 |
| 04 | -38.42, -13.30, 91.88 | -38.29, -13.29, 91.43 | -39.72, -13.66, 89.93 |
| 05 | -32.10, -48.92, 54.99 | -31.32, -48.59, 54.41 | -29.17, -48.56, 53.36 |
| 06 | -28.12, -5.36, 64.13 | -28.28, -5.37, 64.42 | -24.58, -1.25, 66.35 |
| 07 | -36.53, -3.82, 52.77 | -35.75, -4.06, 52.20 | -38.96, -6.81, 54.15 |
| 08 | -32.99, -24.39, 48.69 | -32.72, -24.79, 48.2 | -33.56, -24.44, 48.71 |
| 09 | -39.51, -24.77, 65.75 | -39.46, -24.82, 64.83 | -40.05, -23.80, 65.28 |
| 10 | -39.23, -23.90, 59.8 | -42.42, -24.80, 57.15 | -44.75, -21.48, 54.45 |

***Note:*** The table presents subject-wise $R^2$ maximum coordinates $(x, y, z)$ for three activation models (Magnitude, Neuron, and Cosine). Data corresponds to **Dataset 2**.

**Table S5.** Experimental validation across the three activation models and four control sites

| MEP amplitudes (mV) across targets | | | | | | | |
|---|---|---|---|---|---|---|---|
| Sub No. | Magnitude | Neuron | Cosine | Anterior | Posterior | Superior | Inferior |
| 01 | 0.856 | 0.779 | 0.648 | 0.373 | 0.075 | 0.429 | 0.303 |
| 02 | 0.683 | 0.854 | 0.409 | 0.088 | 0.778 | 0.294 | 0.209 |
| 03 | 0.219 | 0.267 | 0.195 | 0.422 | 0.036 | 0.109 | 0.045 |
| 04 | 0.256 | 0.314 | 0.241 | 0.216 | 0.211 | 0.023 | 0.195 |
| 05 | 0.462 | 0.462 | 0.468 | 0.361 | 0.023 | 0.157 | 0.202 |
| 06 | 1.103 | 0.876 | 0.576 | 0.997 | 0.216 | 0.101 | 0.251 |
| 07 | 0.542 | 0.542 | 0.344 | 0.26 | 0.36 | 0.496 | 0.017 |
| 08 | 1.440 | 1.440 | 1.074 | 0.281 | 0.753 | 0.864 | 0.287 |
| 09 | 0.466 | 0.466 | 0.169 | 0.162 | 0.351 | 0.684 | 0.054 |
| 10 | 1.346 | 1.325 | 0.892 | 0.453 | 1.057 | 0.815 | 0.618 |
| **Mean** | **0.737** | **0.733** | **0.502** | **0.361** | **0.386** | **0.397** | **0.218** |

***Note***: The table shows subject-wise mean MEP peak-to-peak amplitudes across three activation models (Magnitude, Neuron, and Cosine) and four controls sites (Anterior, Posterior, Superior, and Inferior). Results indicate that the magnitude and neuron models yielded significantly higher MEPs compared to the cosine model and the four surrounding control sites. These findings suggest better localization of cortical muscle representations for the Magnitude and Neuron activation models. Data corresponds to **Dataset 2**.

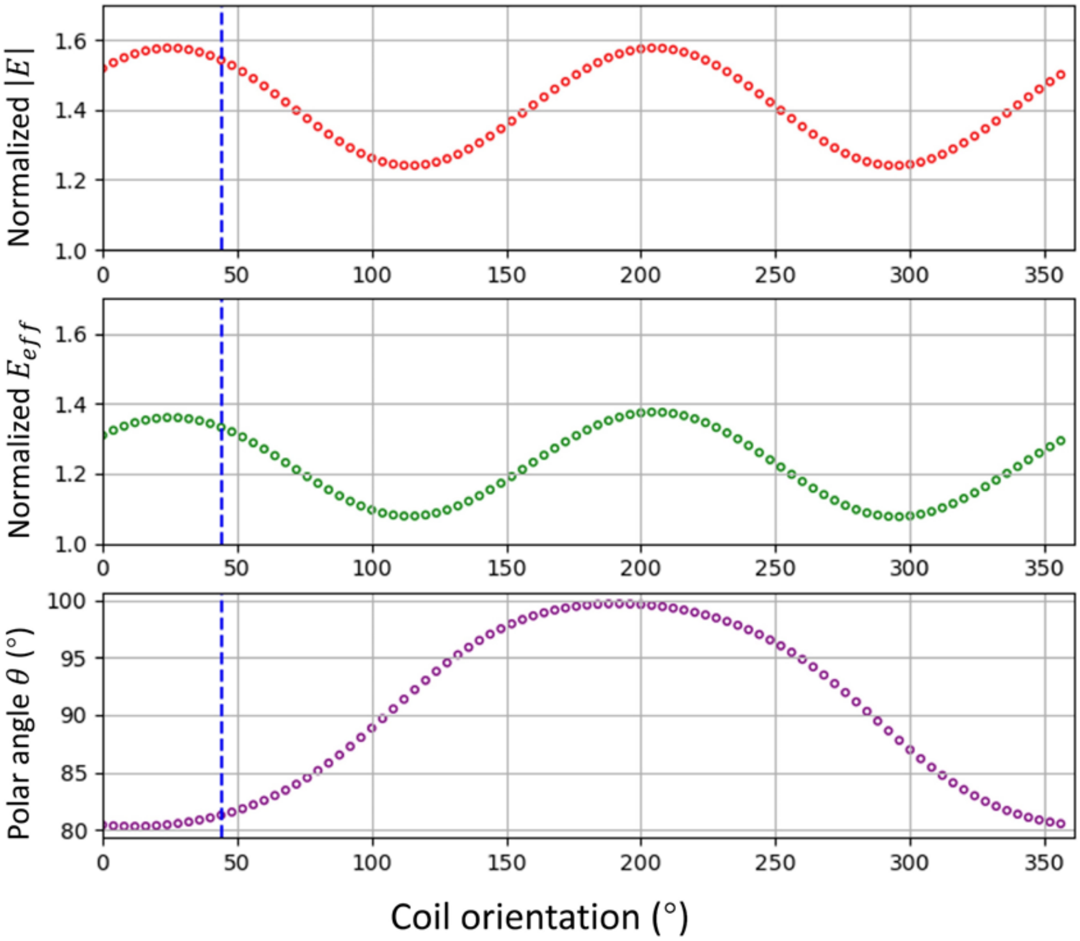


Figure S2. Simulated variations in E-field magnitude $|E|$, effective E-field $E_{eff}$, and polar angle $\theta$ across coil orientations from 0° to 360°. The 0° orientation corresponds to the posterior-to-anterior (PA) direction, aligned with the mid-sagittal line. The PA 45° position is indicated by a blue vertical dashed line. The y-axis in the $|E|$ and $E_{eff}$ plots represents normalized values. Coil orientation was changed in 4° increments.
